# Changes in Rumen Microbial Profiles and Subcutaneous Fat Composition When Feeding Extruded Flaxseed Mixed With or Before Hay

**DOI:** 10.3389/fmicb.2018.01055

**Published:** 2018-05-25

**Authors:** Renee M. Petri, Payam Vahmani, Hee Eun Yang, Michael E. R. Dugan, Tim A. McAllister

**Affiliations:** ^1^Department for Farm Animals and Veterinary Public Health, Institute of Animal Nutrition and Functional Plant Compounds, University of Veterinary Medicine, Vienna, Austria; ^2^Lacombe Research and Development Centre, Agriculture and Agri-Food Canada, Lacombe, AB, Canada; ^3^Lethbridge Research and Development Centre, Agriculture and Agri-Food Canada, Lethbridge, AB, Canada

**Keywords:** rumen, CLA, vaccenic acid, DNA, bacteria, 16S rRNA, flaxseed, biohydrogenation

## Abstract

Extruded flaxseed (25%) and ground hay (75%) were each fed (DM basis) either together in a total mixed ration (TMR) or as flaxseed first followed by hay (non-TMR) to three pens of eight crossbred steers (*n* = 24 per diet) for 240 days. Compared to TMR, feeding non-TMR enriched subcutaneous fat with α-linolenic acid (ALA, 18:3n-3) and its biohydrogenation intermediates including vaccenic acid [*trans*(*t*)11-18:1], rumenic acid [*cis*(*c*)9,*t*11-conjugated linoleic acid] and conjugated linolenic acid (CLnA). Rumen microbial analysis using QIIME indicated that 14 genera differed (*P* ≤ 0.05) between TMR and the non-TMR. *Azoarcus* and *Streptococcus* were the only genera which increased in relative abundance in the TMR fed steers, whereas *Methanimicrococcus, Moryella, Prevotella, Succiniclasticum, Succinivibrio, Suttenella*, and *TG5* decreased as compared to steers fed the non-TMR. Among these, *Moryella, Succiniclasticum*, and *Succinivibrio*, spp. were correlated with fatty acid profiles, specifically intermediates believed to be components of the major biohydrogenation pathway for ALA (i.e., *t*11, *c*15-18:2, *c*9, *t*11, *c*15-18:3, and total CLnA). In addition, negative correlations were found between the less abundant *Ruminoccocus-*like OTU60 and major ALA biohydrogenation intermediates, as well as positive correlations with several intermediates from alternative pathways that did not involve the formation of *trans* 11 double bonds. The present results suggest a number of pathways for ALA biohydrogenation are operating concurrently in the rumen, with their balance being influenced by diet and driven by less abundant species rather than members of the core bacterial population.

## Introduction

Most rumen microbes are sensitive to dietary polyunsaturated fatty acids (PUFA) and detoxify them through biohydrogenation (Jenkins et al., 2008). However, not all unsaturated bonds are reduced, enabling some biohydrogenation intermediates (BHI) to escape the rumen, be absorbed in the lower gut and incorporated into body tissues. Over 40 BHI have been identified in ruminant fats including many *trans*(*t)*-18:1, conjugated linoleic acid (CLA), conjugated linolenic acid (CLnA), and non-conjugated/non-methylene interrupted (i.e., atypical) diene isomers (Vahmani et al., 2015). When feeding forage based diets, major BHI typically include *t*-11-18:1(vaccenic acid; VA) and *cis*(*c*)9,*t*11-CLA (rumenic acid, RA), which may have protective effects against cancer, inflammatory diseases, type II diabetes and post-menopausal osteoporosis (Benjamin and Spener, 2009; Field et al., 2009). Diets containing large amounts of rapidly fermented carbohydrate increase the rate of volatile fatty acid production in the rumen. As a result, rumen pH is reduced, which is accompanied by a shift from *t*11-18:1 to *t*10-18:1 containing biohydrogenation pathways. Trans 18:1 has been associated with up-regulation of fat and cholesterol synthesis in cell culture (Vahmani et al., 2017b) and negative effects on blood lipid profiles in animal models (Roy et al., 2007). Effects of many individual BHI have not been studied, and cattle feeding studies have focused on trying to increase VA and RA in beef, while reducing the concentration of other BHI with negative or unknown health effects (Dugan et al., 2011; Vahmani et al., 2015).

Rumen bacteria capable of biohydrogenating dietary PUFA have been previously isolated and studied (Jenkins et al., 2008). *Butyrivibrio fibrisolvens* is a fiber fermenting bacterium known for its ability to isomerize 18:2n-6 (linoleic acid) to RA, and for its further biohydrogenation to VA (Wallace et al., 2007). Likewise, *Megasphaera elsdenii* is a lactate utilizer and is associated with the production of *t*10-18:1 (Klieve et al., 2003). However, the bacteria which are responsible for the final step in biohydrogenation (i.e., from *t*-18:1 to 18:0), and how these bacteria interact *in vivo* under variable dietary conditions is largely unknown. Previously we found feeding flaxseed as a source of PUFA in forage-based diets increases VA and RA in beef, but results have been variable (Nassu et al., 2011; Mapiye et al., 2013a,b). In one study, we found 9 species of rumen bacteria were associated with high or low levels of VA in beef adipose tissue (Petri et al., 2014). In another study, where higher levels of VA and RA were found (Mapiye et al., 2013b), we anecdotally observed steers may have been sorting dietary components and consuming flaxseed before forage (red clover silage). The preferential consumption of flaxseed would have resulted in high concentrations of PUFA entering the rumen in a shorter period of time, and under *in vitro* conditions, high PUFA levels have been shown to increase VA and RA in rumen fluid (Beam et al., 2000; Troegeler-Meynadier et al., 2003). We recently tested and confirmed the hypothesis that feeding extruded flaxseed prior to hay, as opposed to in a total mixed ration (TMR), would lead to substantial increases in VA and RA in steer erythrocytes (Vahmani et al., 2016c), muscle (Vahmani et al., 2016b), and adipose tissue (Vahmani et al., 2017a). The objectives of the present study were to determine how rumen bacterial profiles and adipose tissue fatty acid profiles of growing cattle would be affected by feeding extruded flaxseed either before hay (non-total mixed ration, non-TMR) or together with hay (TMR). Specifically we aimed to determine which bacterial species would be associated with higher or low levels of BHI in beef adipose tissue, and their similarity to biohydrogenation associated bacterial species previously associated with beef with high or low VA content. We hypothesized that the feeding of hay after the consumption of extruded flaxseed would shift microbial fermentation, the abundance of microbes associated with biohydrogenation and the fatty acid profile of the adipose tissue.

## Materials and methods

### Animals and diets

Animal management, diets and experimental design were previously described (Vahmani et al., 2016b) and ethical experimental practices were reviewed and approved by Lacombe Research and Development Centre (LaRDC) Animal Care Committee (CCAC, 2009). Briefly, 48 Angus cross steers (325.2 ± 15.9 kg; 10.4 ± 0.5 months old) were stratified by weight and randomly assigned to 6 pens containing 8 steers in each pen. Pens were then randomized to either TMR or non-TMR with three pens per treatment. The TMR included a commercial extruded flaxseed product (Supplemental Table 1; 25% of DM; linPRO-R™, O&T Farms, Regina, SK), and a vitamin supplement (0.25% of DM providing 2200, 440, and 135 IU of vitamin A, D3, and E per kg of diet), which were mixed together daily with tub ground timothy hay (alfalfa/grass hay; 74.75% of diet DM) and fed once daily at 08:30 h. The non-TMR treatment included the same ingredients in the same proportions as the TMR, but linPRO-R™ (mixed with vitamin supplement) was fed on its own at 08:30 h followed by tub ground hay at 11:30 h. The linPRO-R™ contained co-extruded flaxseed (50%), field peas (40%), and alfalfa (10%). On a DM basis, diets were formulated to provide 16.6% crude protein, 7.5% crude fat, 29.7% acid detergent fiber, 1.26% Ca, 0.24% P, and 3 MCal of digestible energy per kg. The steers were provided free choice trace mineralized salt (96.5% NaCl, 4,000 mg/kg Zn, 1,600 mg/kg Fe, 1,200 mg/kg Mn, 330 mg/kg Cu, 100 mg/kg I, and 40 mg/kg Co). The major fatty acids (% of total fatty acids) in the diet were α-linolenic acid (ALA; 45.9%), linoleic acid (18.1%) and oleic acid (18.2%). Steers in each pen were group fed to appetite for the remainder of the day with hay and had free access to water. For the non-TMR treatment, all linPRO-R™ was readily consumed before the hay was fed.

### Sample collection

Steers (*n* = 48) were slaughtered at the LaRDC abattoir over four slaughter dates in October 2015 (two steers/pen/diet/slaughter day) at an average of 242 days (based on slaughter date) on feed with an average backfat depth of 13 mm between the 12th and 13th rib over the right *longissimus thoracis* muscle. Steers were maintained on diets until slaughter. Steers were stunned, exsanguinated and dressed in a commercial manner. At ~20 min post-mortem, samples of subcutaneous fat adjacent to the 12th rib were collected and stored at −80°C until analyzed for FA. At 30 min post-mortem, the rumen was opened and the ruminal contents collected and thoroughly mixed. Sampling time of rumen contents occured at slaughter, 12 h after feed removal. Samples of ruminal contents (solids and fluid) were taken from mid-ventral region of the rumen (250 g) and placed into an open 2 L plastic container. Samples were then hand-mixed, subsampled and put in 50 ml plastic culture tubes. The tubes were flash frozen in liquid nitrogen and stored at −80°C prior to bacterial DNA extraction.

### Bacterial DNA extraction, sequencing, and quantification

Thirty-six samples were randomly selected over the 4 slaughter dates (*n* = 18 per treatment) for DNA extraction. Subsamples were taken (50 mL) from both the rumen liquid and solid digesta, freeze-dried and ball-ground using a Tissue Lyzer (Qiagen, Hilden, Germany). DNA isolation was performed using the Qiagen QIAamp DNA stool mini kit with minor modifications (Qiagen, Hilden, Germany). This included bead beating using a bead-beating homogenizer (B. Braun, Melsungen AG, Germany) for 3 min at maximum amplitude to dissociate microbes from feed particles and to disrupt bacterial cells (Yu and Morrison, 2004). Afterwards, samples underwent chemical removal of cell debris and PCR inhibitors and column based isolation of total genomic DNA according to the manufacturer's instructions. A final elution of 100 μL was obtained. The isolated DNA concentration for each sample was determined using a NanoDrop 2000 (ThermoScientific, Wilmington, DE, USA), and samples were stored at −20°C until sequenced.

One 20 μL aliquot of DNA extract from each sample was sent for amplicon sequencing using a MiSeq Illumina sequencing platform and paired-end technology MR DNA (Shallowater, TX, USA). Sequencing targeted V4 of hypervariable region of the 16S rRNA gene using the primer set 515F (5′-GTGCCAGCMGCCGCGGTAA-3′) and 806R (5′-GGACTACHVGGGTWTCTAAT-3′) to generate an amplicon size of ~300 bp (Caporaso et al., 2011). Libraries were constructed by ligating sequencing adapters and indices onto purified PCR products using the Nextera XT Sample Preparation Kit (Illumina, San Diego, CA, USA) according to the manufacturer's recommendations. Equimolar amounts of each of the libraries were pooled and submitted for sequencing on an Illumina MiSeq Personal Sequencer using a 300 bp read length paired-end protocol. Paired-end reads were merged using fastq-join with a minimum overlap of 200 bp and a maximum difference of 3 percent (Aronesty, 2013).

Sequence quality control and analyses were performed using the QIIME pipeline (Caporaso et al., 2010). Sequences were first quality filtered following previously published recommendations (Caporaso et al., 2012; Bokulich et al., 2013) and then screened for chimeras using the UCHIME algorithm (Edgar et al., 2011) implemented in USEARCH (version 6.1544; 21). The remaining high quality 16S rRNA gene sequences were clustered into operational taxonomic units (OTUs) at 97% similarity using the *de novo* reference OTU picking method and USEARCH (version 6.1544). PyNAST (Caporaso et al., 2011) was used to align the representative sequences for each OTU and a phylogenetic tree was created using FastTree (Price et al., 2010) and taxonomy with the RDP classifier (Wang et al., 2007) against the Greengenes database (gg_97_otus_4feb2011.fasta; McDonald et al., 2012). The degree of similarity between sequences was defined as 97% to obtain OTU identity at the species level. A total of 7,585,132 sequences clustered into 1,030 OTUs for further analysis. All OTUs with a relative abundance >0.1% (165 OTUs) were included in the statistical analysis. For calculation of the nonparametric species richness estimators, Chao 1 and the Shannon and Simpson diversity indices were determined using QIIME, with sample rarefaction set at 110,073 sequences per sample based on the sample with the least number of sequences. Principal coordinate analysis (PCoA) beta-diversity plots were built using Bray Curtis analysis in QIIME (Caporaso et al., 2012).

Sequencing data are available in the GenBank database under the accession number SAMN08455775.

### Subcutaneous fatty acid analysis

Subcutaneous fat samples (50 mg) were freeze-dried and direct methylated with sodium methoxide (Aldai et al., 2009). Internal standard, 1 ml of 1 mg *c*10-17:1 methyl ester/ml hexane (standard no. U-42M form Nu-Check Prep Inc., Elysian, MN, USA) was added prior to adding methylating reagent. Fatty acid methyl esters (FAME) were analyzed using a CP-3800 gas chromatograph (GC) equipped with an 8600-series autosampler (Varian Inc., Walnut Creek, CA, USA). Most FAME were analyzed using the 150 and 175°C plateau temperature programs described by Kramer et al. (2008) using a CP-Sil88 column (100 m, 25 μm ID, 0.2 μm film thickness, Agilent Technologies, Santa Clara, CA), except for *t*7, *c*9-18:2, and *c*9, *t*11-18:2, which were analyzed according to Turner et al. (2011) using an SLB IL 111 column (30 m, 0.25 mm ID, 0.2 mm film thickness, Supelco Inc., Bellefonte, PA).

Reference standard no. 601 from Nu-Check Prep Inc., (Elysian, MN, USA) was used for identification of most FAME. Branched-chain FAME were identified using the BC-Mix1 standard, (Applied Science, State College, PA, USA). For CLA isomers, the UC-59M standard from Nu-Chek Prep Inc. was used which contains all four positional CLA isomers. *Trans*-18:1, CLA and other BHI not included in the standard mixtures were identified by their retention times and elution orders as reported in the literature (Cruz-Hernandez et al., 2004; Kramer et al., 2008; Gómez-Cortés et al., 2009) and this included the recently identified Δ-9 desaturation products of *t*18:1 isomers (Vahmani et al., 2016a). The FAME were quantified using chromatographic peak area and internal standard based calculations as described in Vahmani et al. (2016b).

### Statistical analysis

Fatty acid profiles and percent abundance of bacterial populations were analyzed with the mixed models procedure of SAS (2009) using a completely randomized design with diet as the main effect and pen as a random effect. Treatment means were generated and separated using the LSMEANS and PDIFF options, respectively (SAS, 2009). Analysis of variance for sequences and diversity indices was performed using the Shapiro-Wilk Test in Proc Univariate (SAS), almost all parameters were significant, indicating that the data was not normally distributed. To relate rumen bacterial profiles to fatty acid profiles, percent abundance of genus level taxa were additionally analyzed using Spearman's rank order correlation to fatty acid data, using the Proc Corr procedure of SAS (2009). All data reported are least square means with standard error of the mean. Differences were declared significant at *P* < 0.05 and tendencies declared at 0.05 < *P* < 0.10.

## Results

### Animal performance

Data on animal performance are detailed in Vahmani et al. (2016b). In summary, steers fed non-TMR had slightly lower dry matter intake than those fed TMR (10.6 vs. 11.4 kg/d; *P* = 0.02), but average daily gain, final live weight and backfat thickness (overall means: 1.18 kg/d, 610 kg and 12.3 mm respectively) did not differ (*P* > 0.10).

### Subcutaneous fatty acid profiles

The proportions of total PUFA and n-3 PUFA in subcutaneous fat were greater (*P* < 0.05) in steers fed non-TMR than those fed TMR (Table 1). Feeding non-TMR also increased (*P* < 0.05) the proportions of individual n-3 PUFA including ALA and 20:4n-3, and tended to increase (*P* = 0.09) the proportion of 20:3n-3. ALA was the most prominent n-3 PUFA, accounting for more than 87% of total n-3 PUFA in subcutaneous fat from both TMR and non-TMR steers. For n-6 PUFA, only 20:4n-6 was affected by dietary treatments, which tended to be lower (*P* = 0.06) for non-TMR than TMR steers. For both TMR and non-TMR steers, linoleic acid was the most prominent n-6 PUFA, accounting for more than 96% of total n-6 PUFA. Feeding non-TMR increased (*P* < 0.01) the proportions of total CLnA as well as individual CLnA isomers including *c*9,*t*11, *t*15-18:3, and *c*9, *t*11, *c*15-18:3 in subcutaneous fat. *C*9, *t*11, *c*15-18:3 was the main CLnA isomer making up 52 and 56% of total CLnA for TMR and non-TMR steers, respectively.

**Table 1:** Effect of feeding steers extruded flaxseed (linPRO-R™) and hay together as a total mixed ration (TMR), or sequentially (non-TMR) on fatty acid profiles of subcutaneous fat (% of total fatty acids).

**Fatty acid^c^**	**TMR**	**Non-TMR**	**SEM^a^**	***P*-value^b^**
∑ **PUFA**	2.21	2.42	0.049	0.004
∑ **n6-PUFA**	1.17	1.20	0.019	0.26
C18:2n-6	1.13	1.16	0.018	0.27
C20:3n-6	0.03	0.03	0.002	0.52
C20:4n-6	0.020	0.017	0.001	0.06
∑ **n3-PUFA**	1.04	1.23	0.032	<0.001
C18:3n-3	0.91	1.07	0.029	<0.001
C20:3n-3	0.03	0.04	0.002	0.09
C20:4n-3	0.04	0.05	0.003	0.03
C22:5n-3	0.06	0.06	0.005	0.91
∑ **CLnA**	0.52	0.86	0.032	0.002
*c*9,*t*11,*t*15-18:3	0.25	0.36	0.011	0.002
*c*9,*t*11,*c*15-18:3	0.27	0.49	0.023	0.002
∑ **CLA**	2.64	3.78	0.119	0.003
*t*7,*c*9-18:2	0.11	0.10	0.003	0.07
*c*9,*t*11-18:2	1.91	2.69	0.103	0.006
*t*11,*c*13-18:2	0.37	0.77	0.022	<0.001
*t*11,*t*13-18:2	0.12	0.11	0.008	0.60
*t*,*t*-CLA	0.13	0.11	0.004	0.07
∑ **AD**	4.15	4.33	0.085	0.14
*t*11,*t*15-18:2	0.31	0.43	0.017	<0.001
*t*9,*t*12-18:2	0.06	0.05	0.002	0.03
*c*9,*t*14-18:2	0.38	0.29	0.007	<0.001
*c*9,*t*13-18:2	0.50	0.42	0.009	<0.001
*c*9,*t*15-18:2	0.39	0.26	0.009	<0.001
*c*9,*t*12-18:2	0.13	0.10	0.004	0.006
*t*9,*c*12-18:2	0.13	0.16	0.004	<0.001
*t*11,*c*15-18:2	1.87	2.45	0.059	<0.001
*c*9,*c*15-18:2	0.18	0.11	0.005	<0.001
*c*12,*c*15-18:2	0.22	0.08	0.010	<0.001
∑***trans*****-18:1**	9.37	10.30	0.300	0.04
*t*4-18:1	0.02	0.02	0.002	0.20
*t*5-18:1	0.02	0.02	0.002	0.41
*t*6-*t*8-18:1	0.50	0.47	0.016	0.18
*t*9-18:1	0.49	0.47	0.011	0.08
*t*10-18:1	0.59	0.57	0.014	0.43
*t*11-18:1	4.92	6.82	0.202	<0.001
*t*12-18:1	0.59	0.42	0.018	0.003
*t*13-*t*14-18:1	1.31	0.94	0.058	0.01
*t*15-18:1	0.46	0.29	0.023	0.006
*t*16-18:1	0.46	0.29	0.019	0.003
∑***cis*****-MUFA**	41.0	38.8	0.658	0.02
*c*9-14:1	1.37	1.29	0.094	0.57
*c*7-16:1	0.14	0.14	0.003	0.83
*c*9-16:1	3.85	4.01	0.218	0.60
*c*10-16:1	0.23	0.22	0.020	0.74
*c*11-16:1	0.029	0.026	0.002	0.28
*c*9-17:1	0.61	0.60	0.020	0.57
*c*9-18:1	31.9	30.0	0.387	0.002
*c*11-18:1	1.075	1.18	0.044	0.09
*c*12-18:1	0.49	0.25	0.012	<0.001
*c*13-18:1	0.38	0.34	0.021	0.22
*c*14-18:1	0.08	0.06	0.003	0.003
*c*15-18:1	0.52	0.33	0.019	<0.001
*c*16-18:1	0.08	0.05	0.003	0.002
*c*9-20:1	0.12	0.11	0.003	0.30
*c*11-20:1	0.182	0.182	0.006	0.95
∑ **BCFA**	1.86	1.81	0.029	0.22
*iso*-14:0	0.081	0.076	0.003	0.12
*iso*-15:0	0.23	0.22	0.005	0.18
*anteiso*-15:0	0.25	0.24	0.007	0.19
*iso*-16:0	0.27	0.25	0.007	0.17
*iso*-17:0	0.34	0.34	0.005	0.75
*anteiso*-17:0	0.57	0.57	0.007	0.88
*iso*-18:0	0.12	0.12	0.004	0.29
∑ **SFA**	37.6	37.1	0.519	0.483
10:0	0.04	0.04	0.002	0.261
12:0	0.08	0.08	0.003	0.297
14:0	3.48	3.68	0.116	0.235
15:0	0.54	0.49	0.020	0.125
16:0	22.0	22.5	0.306	0.384
17:0	0.72	0.68	0.031	0.494
18:0	10.5	9.4	0.321	0.046
19:0	0.13	0.13	0.004	0.775
20:0	0.09	0.09	0.005	0.739
22:0	0.03	0.03	0.003	0.686

a *Standard error of mean*.

b *P < 0.05 indicates a significant difference between TMR and Non-TMR. ^c^c, cis; t, trans; Σ PUFA, sum of polyunsaturated fatty acids (Σ n-6 + Σ n-3); Σ n-3 = sum of 18:3n-3, 20:5n-3, 22:5n-3; Σ n-6 = sum of 18:2n-6, 20:3n-6, 20:4n-6; ΣCLnA = sum of conjugated linolenic acids (c9,t11,t15-18:3, c9,t11,c15-18:3); Σ CLA = sum of conjugated linoleic acids (t12,t14-, t11,t13-, t10,t12-, t9,t11-, t8,t10-, t7,t9- t6,t8-, c9,t11-, t7,c9-, t11,c13-18:2); Σ AD = sum of atypical 18:2 (t11,t15-, c9,t14-/c9,t13-, c9,t15-, c9,t12-, t9,c12-, t11,c15-, c9,c15-, c12,c15-18:2); Σ t-18:1 = sum of trans-18:1 isomers (t6-, t7-,t8-, t9-, t10-, t11-, t12-, t13-, t14-, t15-, t16-18:1); Σc-MUFA = sum of cis-monounsaturated fatty acids (c9-14:1, c7-16:1, c9-16:1, c11-16:1, c9-17:1, c9-18:1, c11-18:1, c12-18:1, c13-18:1, c14-18:1, c15-18:1, c16-18:1, c9-20:1, c11-20:1); Σ BCFA = sum of branched chain fatty acids (iso-15:0, anteiso15:0, iso16, iso17:0, anteiso17:0, iso18:0); Σ SFA = sum of saturated fatty acids (14:0, 15:0, 16:0, 17:0, 18:0, 19:0, 20:0, 22:0, 24:0)*.

The proportions of total and individual CLA isomers including RA and *t*11, *c*13-18:2 were increased (*P* < 0.01) with non-TMR compared to TMR treatment. RA was the main CLA isomer accounting for 72% of total CLA in subcutaneous fat from both TMR and non-TMR steers. The second major CLA isomer was *t*11, *c*13-18:2, accounting for 14 and 20% of total CLA in subcutaneous fat for TMR and non-TMR steers, respectively.

Feeding the non-TMR increased (*P* < 0.05) the proportions of all 10 atypical diene isomers in subcutaneous fat compared to TMR. The predominant isomer was *t*11, *c*15-18:2 accounting for 45 and 57% of total atypical dienes in subcutaneous fat from TMR and non-TMR steers, respectively. The second major isomer was *c*9, *t*13-18:2 accounting for 12 and 10% of total atypical dienes for TMR and non-TMR steers, respectively. This was followed by *t*11, *t*15-18:2 accounting for 7 and 9% of total atypical dienes for TMR and non-TMR steers, respectively.

Compared to TMR, feeding non-TMR increased (*P* < 0.05) total *t*-18:1 by 10% in subcutaneous fat. The predominant *t*-18:1 isomer was VA accounting for 52 and 66% of total *t*-18:1 in subcutaneous fat from TMR and non-TMR steers, respectively. Feeding non-TMR increased (*P* < 0.01) the proportions of VA, reduced (*P* ≤ 0.01) *t*12- to *t*16-18:1 isomers, tended to reduce (*P* = 0.08) *t*9-18:1, but did not affect the proportions of *t*4- to *t*8-18:1 isomers (Table 1).

Feeding non-TMR decreased (*P* = 0.02) the proportions of total *c*-monounsaturated fatty acids (*c*-MUFA) which was mainly related to the reduction in *c*9-18:1 (oleic acid), the predominant *c*-MUFA in subcutaneous fat. The proportions of minor *c*-18:1 isomers including *c*12-18:1, *c*14-18:1, *c*15-18:1, and *c*16-18:1 were also reduced (*P* < 0.01) with the non-TMR. Conversely, treatment did not affect (*P* > 0.10) concentrations of branched chain fatty acids or SFA except for 18:0, which was reduced (*P* = 0.046) with the non-TMR.

### Bacterial diversity

**Figure 1:**
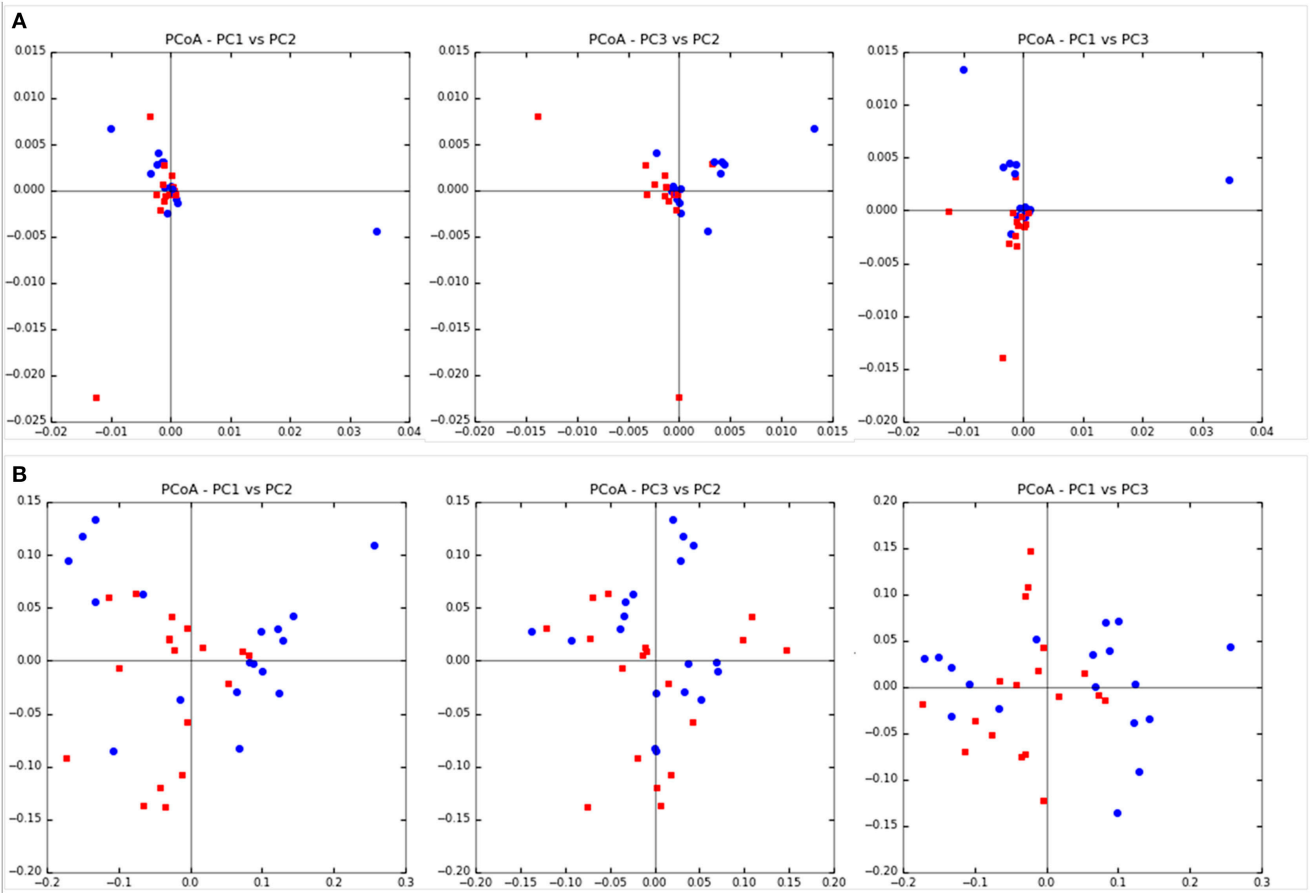
Beta diversity analysis using Unifrac analysis of dietary differences between steers fed extruded flaxseed and hay together (TMR; squares) or sequentially (Non-TMR; circles). **(A)** Unweighted Unifrac analysis with PC1:45.1%, PC2:21.3%, and PC3:14.76%. **(B)** Weighted Unifrac analysis with PC1: 24.3%, PC2:11.27%, and PC3:8.81%.

Community diversity between steers fed TMR vs. those fed non-TMR were not different in the number of observed OTUs, sequencing coverage as per the Good's coverage estimator or the Simpson index of evenness (Table 2). A trend toward increased Shannon's diversity (*P* = 0.09) was seen for TMR fed animals. Beta diversity analysis using weighted and unweighted Unifrac analysis are shown in Figure 1. Unweighted Unifrac analysis showed a larger amount of variation in principal components (PC) 1 and 2 (PC1:45.1%, PC2:21.3%) with respect to the dietary treatments when compared with weighted Unifrac (PC1: 24.3%, PC2:11.27%). However, no distinct clustering based on treatment was evident.

**Table 2:** Rumen bacterial community diversity analysis of steers fed extruded flaxseed with hay (TMR) or sequentially (non-TMR).

**Diversity Indices**	**TMR**	**Non-TMR**	**SEM^a^**	***P*-value^b^**
Number of observed OTUs	1,047	1,047	2.0	0.984
Good's coverage	0.9999	0.9999	0.00003	0.296
Shannon	7.41	7.29	0.048	0.094
Simpson	0.984	0.982	0.0008	0.151

a *Standard error of mean*.

b *P < 0.05 indicates a significant difference between TMR and Non-TMR. P < 0.10 indicates a trend toward significance*.

### Rumen taxonomic classification

**Figure 2:**
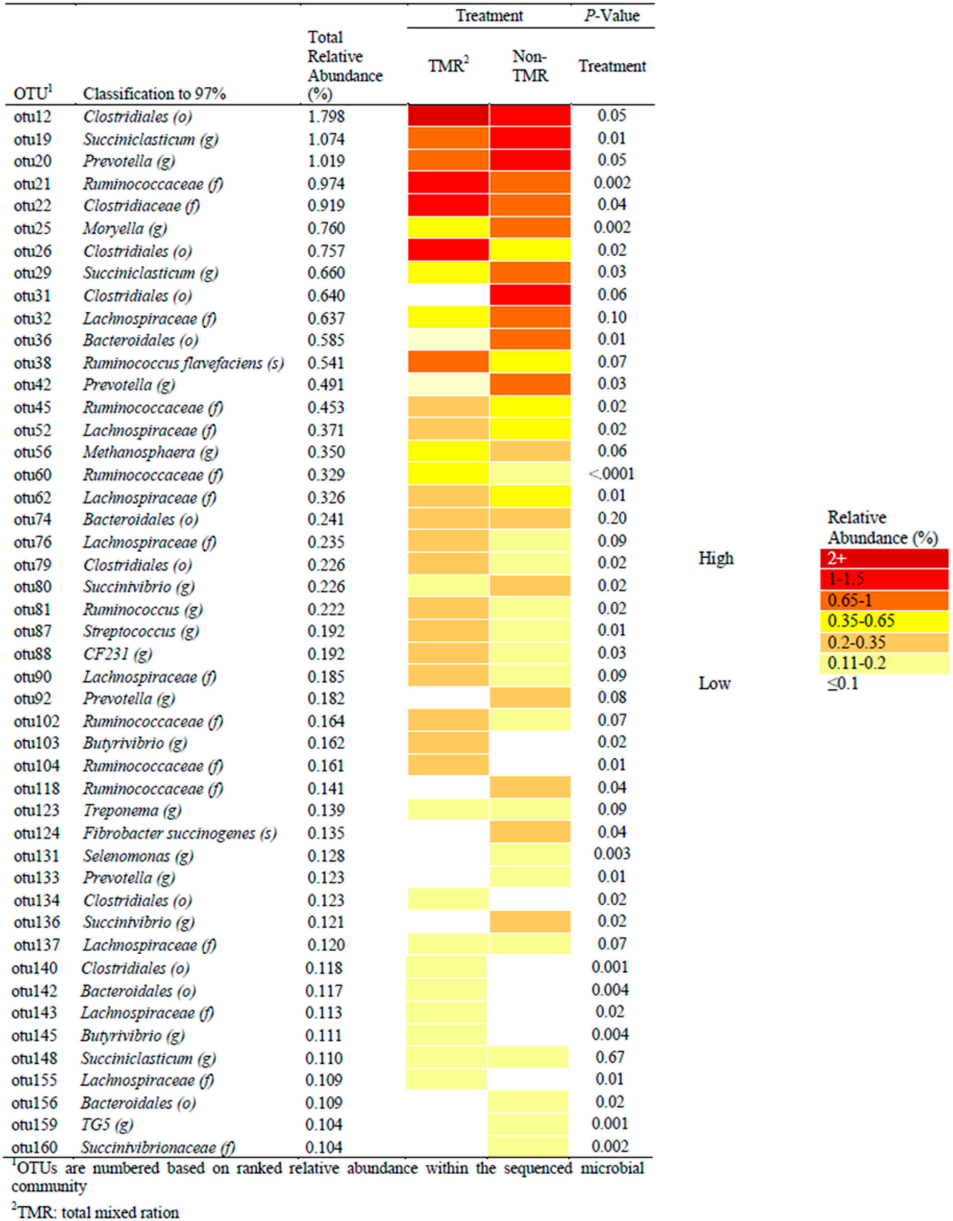
Comparisons of significant OTUs, identified to 97% identity and with relative abundance >0.1%, between steers fed extruded flaxseed and hay together (TMR) or sequentially (non-TMR). Relative abundance of OTUs is indicated by color with dark red having the highest abundance (>2.0%) and white having the lowest relative abundance (< 0.1%) of the total sequenced population.

Sequence alignment to the Greengenes database provided identification of a number of OTUs to the genera and species taxonomic level (Figure 2). The most abundant OTU (OTU1; 4.66% relative abundance) was identified as a *Methanobrevibacter* spp. from the kingdom Archaea, which was not affected by treatment (i.e., TMR vs. non-TMR; S1). The average relative abundance of Archaea was between 5.5 and 8.9% of the OTU population, with no significant difference between treatments. Among OTUs affected by diet and identifiable to the genera level, *Succiniclasticum*-like OTU19 was more abundant when feeding the non-TMR. Similar to OTU19, OTUs 20, 42, 92, and 133, all of which mapped to *Prevotella* increased in total relative abundance when feeding the non-TMR. In contrast, all OTUs identified as *Ruminococcus*-like species, with the exception of OTU118, were found at higher levels when feeding the TMR. Only two OTUs were identified to the species level, including OTU38 as *Ruminococcus flavefaciens* and OTU124 as *Fibrobacter succinogenes*. Despite both species being common rumen cellulolytic bacteria, they showed varying responses to diet, with relative abundance of *Fibrobacter succinogenes*-like species increasing, while *Ruminococcus flavefaciens*-like species decreased when feeding the non-TMR.

Statistical analysis of all bacterial genera identified through taxonomic alignment resulted in 14 genera with a significant or trend toward significant difference between steers fed TMR vs. non-TMR diets (Table 3). Genera *Azoarcus* and *Streptococcus* were the only genera which were increased in TMR fed steers, whereas *Methanimicrococcus, Moryella, Prevotella, Succiniclasticum, Succinivibrio, Suttenella*, and *TG5* increased in relative abundance in steers fed the non-TMR.

**Table 3:** Effect of feeding steers extruded flaxseed (linPRO-R™) and hay together as a total mixed ration (TMR), or sequentially (non-TMR) on percent abundance of bacterial genera in the rumen.

**Genus^a^**	**TMR**	**Non-TMR**	**SEM^b^**	***P*-value**
*Azoarcus*	0.05	0.03	0.004	0.02
*Fibrobacter*	0.02	0.48	0.167	0.06
*Methanimicrococcus*	0.05	0.09	0.013	0.02
*Methanosphaera*	0.45	0.37	0.032	0.10
*Moryella*	0.85	1.25	0.092	0.005
*Prevotella*	4.62	6.12	0.526	0.05
*Ruminococcus*	9.12	7.69	0.569	0.09
*Shuttleworthia*	0.13	0.11	0.008	0.07
*Streptococcus*	0.32	0.17	0.033	0.002
*Succiniclasticum*	1.52	2.17	0.150	0.005
*Succinivibrio*	0.15	0.62	0.127	0.01
*Suttonella*	0.04	0.06	0.004	0.02
*TG5*	0.10	0.16	0.013	0.002
*YRC22*	0.19	0.27	0.029	0.09

a *Genera shown represent those that were significantly different (P < 0.05) or tended to be different (P < 0.10) between TMR and non-TMR*.

b *Standard error of mean*.

### Microbes and fatty acid correlations

Spearman's rank correlations of genera impacted by diet were used to explore their relation to subcutaneous fatty acids (Table 4). Of the 14 genera analyzed, 8 correlated (*P* ≤ 0.05) to *t*11, *c*15-18:2, *c*9,*t*11, *c*15-18:3, and total CLnA. *Azoarcus, Methanosphaera, Shuttelworthia*, and *Streptococcus* spp. were all negatively correlated with *t*11, *c*15-18:2, *c*9,*t*11, *c*15-18:3, and total CLnA, with *Streptococcus* showing the strongest correlation (*r*^2^ = 0.46) to *c*9, *t*11, *c*15-18:3. In contrast, *Moryella, Succiniclasticum* and *Succinivibrio* spp. were all positively correlated with *t*11, *c*15-18:2, *c*9,*t*11, *c*15-18:3, and total CLnA. The strongest correlation was between *Succinivibrio* and *c*9, *t*11, *c*15-18:3 (*r*^2^ = 0.32).

**Table 4:** Spearman's rank order correlation of bacterial genera to subcutaneous fatty acids^a^.

**Genera Taxa**		**Fatty acids**		
		***t*11,*c*15,*t*10*c*15-18:2**	***c*9,*t*11,*c*15-18:3**	**CLnA^b^**
*Azoarcus*	cc^c^	−0.40	−0.38	−0.39
	*P*-value	0.02	0.02	0.02
*Methanosphaera*	cc	−0.52	−0.48	−0.44
	*P*-value	0.001	0.003	0.008
*Moryella*	cc	0.42	0.38	0.39
	*P*-value	0.01	0.02	0.02
*Shuttleworthia*	cc	−0.37	−0.34	−0.36
	*P*-value	0.03	0.04	0.03
*Streptococcus*	cc	−0.62	−0.68	−0.66
	*P*-value	<0.001	<0.001	<0.001
*Succiniclasticum*	cc	0.38	0.38	0.37
	*P*-value	0.02	0.02	0.03
*Succinivibrio*	cc	0.52	0.57	0.54
	*P*-value	0.001	<0.001	<0.001
*TG5*	cc	0.41	0.34	0.33
	*P*-value	0.01	0.05	0.05

a *Only correlations with P < 0.10 are presented*.

b *Sum of conjugated linolenic acids (c9,t11,t15-18:3, c9,t11,c15-18:3)*.

c *Correlation coefficient*.

Among the OTUs that were affected by treatment (TMR vs. non-TMR), the relative abundance of *Ruminococcaceae*-like OTU60 correlated (*P* < 0.05) with 42 different fatty acids in subcutaneous fat (Table 5). Out of these only *c*12-18:1, *c*14-18:1, *t*12-18:1, *t*15-18:1, *t*16-18:1, *t*11, *c*13-18:2, *c*9, *t*11, *c*15-18:3, and *c*12, *c*15-18:2 showed moderate correlation coefficients (*r*^2^ ≥ 0.50; *P* < 0.001).

**Table 5:** Correlation of *Ruminococcaceae*- like OTU60 to biohydrogenation intermediates in backfat with a significance of *P* ≤ 0.05.

	**Correlation Coefficient (r^2^)**
**Fatty acid**	**Backfat**
*c*12-18:1	0.56
*c*14-18:1	0.56
*c*15-18:1	0.35
*c*16-18:1	0.47
*t*12-18:1	0.54
*t*15-18:1	0.52
*t*16-18:1	0.53
*t*8,*c*12-18:2	0.46
*t*9,*c*12-18:2	0.42
*t*11*c*13-18:2	0.50
*c*12,*c*15-18:2	0.53
*c*9,*c*15-18:2	0.46
*c*9,*t*11,*c*15-18:3	0.60
*t*11,*c*15	0.49

## Discussion

Several factors have been shown to influence beef fatty acid composition when feeding lipid supplements including amount, type and processing of the lipid supplement, forage to concentrate ratio and forage type (Vahmani et al., 2015). In this study, we found the timing of lipid supplement feeding (i.e., TMR vs. non-TMR) can remarkably effect subcutaneous fatty acid composition, even though diets had identical processing (extrusion) and chemical composition. The main fatty acids in subcutaneous fat that were increased with the non-TMR included n-3 PUFA and BHI including CLnA, CLA, AD, and *t*-18:1.

The increased levels of n-3 PUFA in subcutaneous fat of steers fed non-TMR were mainly related to increased ALA, the major fatty acid in linPRO-R™. This signifies increased by-pass of ALA from the rumen when linPRO-R™ was fed on its own prior to hay (i.e., non-TMR). The ALA level in subcutaneous fat in the present study was comparable to Mapiye et al. (2013b) who fed 15% flaxseed in a red clover silage-based diet, but was greater than found by Nassu et al. (2011) (0.70–0.80%), who fed 15% flaxseed in barley silage or hay-based diets.

Feeding the Non-TMR increased CLnA by 64% over TMR, mainly in the form of *c*9, *t*11, *c*15-18:3, with levels exceeding reports from previous flaxseed feeding trials (Nassu et al., 2011; Mapiye et al., 2013a,b). It is noteworthy that several studies have demonstrated CLnA has anti-inflammatory, immune-modulatory, anti-obesity, and anti-carcinogenic properties in cell culture and animal models (Hennessy et al., 2011), and may improve the healthfulness of beef.

Consistent with the CLnA response, CLA levels also increased with the non-TMR, mainly as a result of increases in *c*9, *t*11-18:2, and *t*11, *c*13-18:2. The higher levels of RA in subcutaneous fat from non-TMR steers is consistent with increased levels of VA, which is the precursor for RA. In fact, the majority of RA in beef tissues originates from Δ-9 desaturation of absorbed VA (Griinari et al., 2000). Both VA and RA have been of targeted interest for enrichment in beef fat because of their purported health benefits (Benjamin and Spener, 2009; Field et al., 2009). The levels of *c*9, *t*11-18:2 in subcutaneous fat from non-TMR fed steers was comparable to earlier studies when feeding 15% flaxseed in forage-based diets (Nassu et al., 2011; Mapiye et al., 2013b), but levels of *t*11, *c*13-18:2 were 2-3 fold more in the present experiment (i.e., with non-TMR).

The increased levels of total atypical dienes in subcutaneous fat from non-TMR steers was consistent with increases in CLA and CLnA. Responses were mainly related to increased levels of *t*11, *c*15-, and *t*11, *t*15-18:2, which were greater than reported in previous studies feeding 10–15% flaxseed in forage-based diets (Noci et al., 2007; Nassu et al., 2011; Mapiye et al., 2013a,b). In contrast, levels of minor atypical dienes including *c*9, *t*12-, *c*9, *t*13-, *c*9, *t*14-, *c*9, and *t*15-18:2 were reduced in non-TMR steers, which is consistent with reduced levels of their precursors (i.e., *t*12-, *t*13-, *t*14-, and *t*15-18:1) in subcutaneous fat. The predominant atypical diene found in flaxseed-fed beef is *t*11, *c*15-18:2, which is generated from reduction of *c*9, *t*11, *c*15-CLnA during ruminal biohydrogenation of ALA (Alves and Bessa, 2014). Further isomerisation of *t*11, *c*15-18:2 may then lead to *t*11, *t*15-18:2, and although the direct health effects of *t*11, *c*15-, and *t*11, *t*15-18:2 are unknown, their Δ-9 desaturation to CLnA may be possible, and consequently be of some health benefit (Hennessy et al., 2011).

Increases in BHI with two and three double bonds for the non-TMR treatment resulted in increased proportions of *t*-18:1 in subcutaneous fat, mainly in the form of VA. The concentration of VA in subcutaneous fatty acids reached 6.8% for the non-TMR treatment, which is comparable to previous findings when preferential consumption of flaxseed over forage was observed (Mapiye et al., 2013b), or when grazing heifers were supplemented with flaxseed once daily on pasture (Noci et al., 2007). In contrast to VA, the concentrations of *t*-18:1 isomers with double bonds from carbon 12 to16 were reduced when feeding the non-TMR, suggesting a shift toward biohydrogenation pathways with intermediates containing *t*-11 bonds. The present study shows that sequential feeding of PUFA source followed by forage (i.e., non-TMR) is an effective way to enrich beef with VA while reducing other *t*-18:1 isomers. In contrast, other *t*-18:1 isomers are either considered detrimental (e.g., *t*9- and *t*10-18:1) or have unknown health effects (e.g., *t*12-16 18:1), making the observed reduction in beef in our study a desirable outcome (Dugan et al., 2011; Vahmani et al., 2015).

The reduced concentration of *c*9-18:1 (oleic acid) in subcutaneous fat with the non-TMR could be related to decreased levels of its precursor, 18:0 (stearic acid). Stearic acid is the main end product of ruminal biohydrogenation of 18-carbon PUFA (Jenkins et al., 2008) and can be delta-9 desaturated to oleic acid in tissues. The reduced concentration of 18:0 suggests incomplete biohydrogenation, which is consistent with the greater concentrations of BHI including *t*-18:1, atypical dienes, CLA and CLnA in subcutaneous fat from non-TMR steers. The reduced concentrations of minor *c*-18:1 with double bonds from carbon 12 to 16 in non-TMR steers was consistent with the response of their *trans* isomers equivalents (i.e., *t*12-*t*16-18:1). This further confirms a shift in biohydrogenation pathways away from minor BHI to those with *t*11 double bonds with the non-TMR treatment.

PUFA are toxic to rumen microbes, and consequently higher amounts of PUFA (i.e., ALA) entering the rumen as a bolus in non-TMR steers would be expected to have a greater impact on microbial populations. However, microbial diversity both within (alpha diversity) and between samples (beta diversity) was largely unaffected by diet. Limited differences in microbial diversity could be in part due to sampling of rumen contents at slaughter, 12 h after feed removal, and therefore changes in relative abundance related directly to the timing of feeding could not be accurately assessed. However, consistent with our findings, Huws et al. (2015) and Castillo-Lopez et al. (2017) showed no changes in the microbial diversity of the rumen bacteria with inclusion of flaxseed in the diet of steers.

Interestingly, those OTU's which showed a significant change in relative abundance with relation to the feeding of TMR or Non-TMR were predominantly members of (9 out of 10) the *Firmicutes, Clostridia*, and *Clostridiales*. However, despite the similarity in phylogeny, half of these OTUs increased in the non-TMR diet and half decreased. Despite published literature indicating that dietary PUFA content inhibits methanogens and protozoa (Sauvant and Giger-Reverdin, 2007; Hook et al., 2010), the most abundant OTU found in the present study was *Methanobrevibacter*-like spp. from the kingdom Archaea (97% identity). The relative abundance of Archaea in this study is similar, but slightly lower, compared to previous published using metagenome analysis and higher than the qPCR Archaea domain abundance (Wallace et al., 2015). However, this is likely due to the primers and methodologies used for determining relative abundance of DNA within a sample. Kong et al. (2010) also detected *Archaea* in steers fed forage-based diets, but found no change in the proportion of *Archaea* with the addition of flaxseed to TMR. The authors stated that it was possible PUFA supplementation was affecting activity instead of quantity of methanogens. However, others have found that the quantity of methanogens present in the rumen does not necessarily correlate with changes in enteric CH_4_ emissions (Tapio et al., 2017).

Given the concentration of VA in subcutaneous fat increased when feeding the non-TMR, we expected the abundance of bacteria involved in final conversion of VA to 18:0 (i.e., Group B bacteria) to be lower in the non-TMR compared to TMR. The main ruminal species known to be involved in the final step of biohydrogenation (i.e., conversion of VA to 18:0) is *Butyrivibrio proteoclasticus* (Moon et al., 2008). Although, the current study was unable to identify changes at the species level, the abundance of *Butyrivibrio* did not differ between TMR and non-TMR. Similarly, Huws et al. (2010) found feeding steers fish oil, which is known to inhibit conversion of VA to 18:0, did not change the abundance of *Butyrivibrio proteoclasticus* in the rumen. In addition to *Butyrivibrio*, several other bacterial genera have been suggested to be involved in the conversion of VA to 18:0, including *Propionibacteria acnes, Selenomonas ruminantium, Enterococcus faecium, Staphylococcus* sp., *Flavobacterium* sp. and *Streptococcus* (McKain et al., 2010). However, none of these bacterial groups differed between diets in our study, making it almost certain that unknown rumen bacterial species that participate in the biohydrogenation process will be discovered in the future.

Among the 14 genera that differed in the rumen of steers fed TMR vs. non-TMR, three including *Fibrobacter, Prevotella and Ruminococcus*, have been previously reported to be influenced by feeding flaxseed to cattle (Li et al., 2012; Petri et al., 2014). However, in contrast to the present study, Petri et al. (2014) found *Fibrobacter* spp. to be less abundant in the rumen when high levels of VA were found in subcutaneous fat of steers fed flaxseed in a hay-based diet. Also in contrast with our findings, Huws et al. (2015) observed a reduced abundance of *Fibrobacter* spp. and *Prevotella* spp. in the rumen of steers fed grass silage supplemented with flaxseed oil. These results are particularly interesting when looking at the significant difference in DMI between the treatment groups, which impacted NDF intake (Vahmani et al., 2016b). Despite these treatment differences, key fiber fermenting genera were not changed in this study, indicating that feed timing had a greater impact than DM and NDF intake. Recently, we found 9 bacterial genera (*Anaerophaga, Asaccharobacter, Fibrobacter, Guggenheimella, Marvinbryantia, Paludibacter, Pseudosphingobacterium, Pseudozobellia, Syntrophococcus*) that were associated with either higher or lower levels of VA in the subcutaneous fat of steers fed forage-based diets containing flaxseed or sunflower-seed (Petri et al., 2014). However, in the present study, no bacteria correlated with VA levels. As a consequence, the increase in VA with the non-TMR might be due to increased bypass of the fatty acids from the rumen rather than change in bacterial populations. It is also likely that the feeding management in the present study resulted in changes in the fatty acid profiles observed through alteration in the ruminal bypass of other BHI. Sequential feeding of linPRO-R™ followed by forage (i.e., non-TMR) could have resulted in reduced fiber raft formation in the rumen, which in turn would increase the rate of passage of BHI from the rumen, resulting in greater post-ruminal absorption and incorporation into tissues. Furthermore, feeding hay on its own after linPRO-R™ would stimulate rumination and increase saliva production which would also potentially increase the flow of BHI to the lower digestive tract. Conversely, when feeding the linPRO-R™ mixed with hay (TMR), the hay would have increased fiber raft formation, which would result in a longer retention time in the rumen, and possibly more complete biohydrogenation. Moreover, the retention of BHI within the TMR fiber raft would result in more fiber-associated bacteria, such as *B. fibrisolvens*, having access to BHI to complete biohydrogenation through to saturation. In contrast, for the non-TMR treatment the rumen bacteria would be unable to completely biohydrogenate the fatty acids due to a faster rate of passage, which would explain the lack of significant correlations. This is supported by the finding that among the genera whose relative abundance increased with non-TMR, *Moryella* spp. and *Succiniclasticum* spp. have been suggested to be members of core rumen microbiome (Li et al., 2012; Jami et al., 2013; Huws et al., 2015). Between the two, *Succiniclasticum* did not correlate to any of the fatty acids in subcutaneous fat, while *Moryella* spp. was positively correlated (*P* ≤ 0.05) to subcutaneous levels of *t*11, *c*15-18:2, *c*9, *t*11, *c*15-18:3, and total CLnA. Given that *c*9, *t*11, *c*15-18:3, and *t*11, *c*15-18:2 are the first and second intermediates in the biohydrogenation pathway of ALA, *Moryella* spp. may play a role in the principal biohydrogenation pathways for ALA. However, previous studies did not observe any increase in the abundance of the rumen core bacterial population, including *Moryella* spp., when flaxseed was fed to cattle (Petri et al., 2014; Huws et al., 2015; Castillo-Lopez et al., 2017). In contrast, in the present study we found extensive correlations between fatty acids in subcutaneous fat, including several biohydrogenation intermediates of ALA (e.g., several *cis* and *trans* 18:1 isomers, *c*9, *c*15-18:2, *t*11, *c*15-18:2, and *c*9, *t*11, *c*15-18:3) with *Ruminococcus-*like OTU60. The abundance of *Ruminococcus* genus decreased by 2% with Non-TMR fed steers and OTU60 accounted for only 16.5% of the *Ruminococcus* sequences (i.e., not a member of the core microbes). Sequence overlap between OTU60 and *B. proteoclasticus*, a main biohydrogenating species, was measured at 85% similarity. Despite the relatively low abundance of OTU60 and its 16S rRNA dissimilarity with *B. proteoclasticus*, OTU60 might play an important role in ruminal biohydrogenation.

## Conclusions

Results of the present experiment indicate feeding extruded flaxseed and hay sequentially rather than mixed in a TMR can influence amounts and types of ALA biohydrogenation intermediates in the subcutaneous fat of steers. Changes in biohydrogenation intermediate levels were not associated with changes in microbial diversity or abundance of dominant populations, but rather were influenced by less abundant genera, many of which likely remain uncultured in the laboratory. However, the role of the rumen bacteria with relation to the rumen lipodome still remains relatively unknown due to a lack of understanding regarding the metabolism of identified OTUs and the diversity of substrate fermentation at the genera and species level. This lack of knowledge and need for further research is further supported by our results. Therefore, a more extensive and detailed database for genomic identification is also critical in furthering our understanding of the rumen bacterial population and the effect that its diversity can have on the fatty acid composition and quality of beef.

## Author contributions

MD and TM conceived and designed the experiments. PV performed the experiments. HY, RP, and PV analyzed the data. MD and TM contributed reagents, materials, and analysis tools. RP, PV, MD, and TM wrote the paper.

### Conflict of interest statement

The authors declare that the research was conducted in the absence of any commercial or financial relationships that could be construed as a potential conflict of interest.
