# Supplementary material for: Changes in Rumen Microbial Profiles and Subcutaneous Fat Composition When Feeding Extruded Flaxseed Mixed With or Before Hay
Source: Front Microbiol. 2018 May 25;9:1055. doi: 10.3389/fmicb.2018.01055 (PMC5981202; doi:10.3389/fmicb.2018.01055)
Supplement: Supplementary file 1 [file Table_1.DOCX]

**Supplemental Table 1**. Most abundance OTUs and their taxonomic assignment based on the GreenGenes databank.

| #OTU ID | OTU Rank | Abundance (%) | Green Genes Taxonomy^1^ |
| --- | --- | --- | --- |
| denovo7796 | otu1 | 4.66 | k__Archaea; p__Euryarchaeota; c__Methanobacteria; o__Methanobacteriales; f__Methanobacteriaceae; g__Methanobrevibacter; s__ |
| denovo142353 | otu2 | 3.49 | k__Bacteria; p__Firmicutes; c__Clostridia; o__Clostridiales; f__Lachnospiraceae; g__Butyrivibrio; s__ |
| denovo71659 | otu3 | 2.96 | k__Bacteria; p__Firmicutes; c__Clostridia; o__Clostridiales; f__Lachnospiraceae; g__Pseudobutyrivibrio; s__ |
| denovo71282 | otu4 | 2.89 | k__Bacteria; p__Firmicutes; c__Clostridia; o__Clostridiales; f__Lachnospiraceae; g__Butyrivibrio; s__ |
| denovo3140 | otu5 | 2.39 | k__Bacteria; p__Firmicutes; c__Clostridia; o__Clostridiales; f__Ruminococcaceae; g__Ruminococcus |
| denovo122599 | otu6 | 2.30 | k__Bacteria; p__Proteobacteria; c__Gammaproteobacteria; o__Aeromonadales; f__Succinivibrionaceae; g__; s__ |
| denovo124357 | otu7 | 2.16 | k__Bacteria; p__Actinobacteria; c__Coriobacteriia; o__Coriobacteriales; f__Coriobacteriaceae; g__; s__ |
| denovo16661 | otu8 | 1.96 | k__Bacteria; p__Proteobacteria; c__Gammaproteobacteria; o__Aeromonadales; f__Succinivibrionaceae; g__Ruminobacter; s__ |
| denovo113474 | otu9 | 1.92 | k__Bacteria; p__Firmicutes; c__Clostridia; o__Clostridiales; f__[Mogibacteriaceae]; g__Mogibacterium; s__ |
| denovo121037 | otu10 | 1.90 | k__Archaea; p__Euryarchaeota; c__Methanobacteria; o__Methanobacteriales; f__Methanobacteriaceae; g__Methanobrevibacter; s__ |
| denovo97655 | otu11 | 1.82 | k__Bacteria; p__Firmicutes; c__Clostridia; o__Clostridiales; f__Lachnospiraceae; g__Butyrivibrio; s__ |
| denovo32476 | otu12 | 1.80 | k__Bacteria; p__Firmicutes; c__Clostridia; o__Clostridiales; f__; g__; s__ |
| denovo28620 | otu13 | 1.57 | k__Bacteria; p__Bacteroidetes; c__Bacteroidia; o__Bacteroidales; f__; g__; s__ |
| denovo55767 | otu14 | 1.41 | k__Bacteria; p__Firmicutes; c__Clostridia; o__Clostridiales; f__Lachnospiraceae; g__Coprococcus; s__ |
| denovo86788 | otu15 | 1.24 | k__Bacteria; p__Firmicutes; c__Clostridia; o__Clostridiales; f__; g__; s__ |

^1^Taxonomy designated by letters: k: kingdom; p: phyla; c: class; o: order; f: family; g: genus; s: species
